# Pigmentary lesions in eyes with rhegmatogenous retinal detachment with flap tears: a retrospective observational study

**DOI:** 10.1038/s41598-022-16508-5

**Published:** 2022-07-21

**Authors:** Midori Ideyama, Yuki Muraoka, Kentaro Kawai, Masaharu Ishikura, Shin Kadomoto, Naomi Nishigori, Takanori Kameda, Kenji Ishihara, Manabu Miyata, Masahiro Miyake, Tomoaki Murakami, Sotaro Ooto, Akitaka Tsujikawa

**Affiliations:** Department of Ophthalmology and Visual Sciences, Kyoto University Graduate School of Medicine, Sakyo-ku, Kyoto, 606-8507 Japan

**Keywords:** Diseases, Medical research, Risk factors, Signs and symptoms

## Abstract

We included 97 patients with unilateral rhegmatogenous retinal detachment (RRD) with posterior vitreous detachment who underwent vitrectomy, and examined pigmentary lesion (PL) characteristics around the sites of original tears using pre- and postoperative ultra-widefield scanning light ophthalmoscopy, green light fundus autofluorescence (FAF) imaging, and intraoperative digital video. If PL did not involve RRD, we used OCT to preoperatively assess any pathologic changes to the lesion. A total of 116 retinal tears (mean count, 1.2 ± 0.5; range, 1–4 per eye) were observed in the detached retina. Overall, 102 (88%), 63 (54%), 14 (12%), and 25 (22%) tears were accompanied by lattice degeneration (LD) or PL, both LD and PL, only LD, and only PL, respectively. In green FAF images, LD showed normal to mild-hyper fluorescence, whereas all PL showed hypofluorescence. On OCT, PL were located at the RPE level, while choroid abnormalities were unclear. In the retinal areas of 22 eyes, which were not affected by RRD, we observed PL without retinal tears; some were accompanied by vitreous traction and tractional retinal detachment. Pre-, intra-, and post-operative assessments of original flap tears suggested that PL might be a risk factor for RRD, developing alongside or separately from LD.

## Introduction

Rhegmatogenous retinal detachment (RRD) is a representative vitreoretinal disease caused by a hole or flap tear in the retina^1–5^. It is accompanied by a flap (horseshoe-like) tear and is associated with the progression of posterior vitreous detachment (PVD)^6,7^. In general, RRD with a flap tear rarely resolves on its own and requires surgeries in almost all cases^8^. The visual outcomes of RRD have improved since adjuvant agents (e.g., triamcinolone acetonide^9,10^ and perfluorocarbon liquids^11,12^) and a wide-viewing system^13,14^ ecame available. However, visual prognosis is poor in cases of RRD involving the macula, and those with re-detachment, long-term RRD, or RRD progressing to proliferative vitreoretinopathy.

Lattice degeneration (LD) is a retinal lesion associated with RRD^2,6,15–19^ that is implicated in the onset of 20% to 65% of RRD cases^3,4,20–24^. Ophthalmoscopically, a typical case of LD presents with a white-striped appearance and slightly elevated margin^16,17^. Pathological findings revealed that the retina is thin in LD and loses its layered-structure, and that the inner limiting membrane is missing and replaced by retinal glial cells^16^. Concentrated vitreous adheres to the margin of LD, and proliferated glial cells extend into the vitreous. In eyes with these pathological characteristics, the retina can be pulled as PVD progresses, leading to the development of retinal flap tear and RRD^16,17,20,25,26^.

Alongside the wide-viewing systems in vitrectomy, ultra-widefield (UWF) imaging is commonly used in clinical practice^27–30^. These modalities have made it easier than it was before to evaluate and treat peripheral retinal lesions. In our practice, we have noticed high incidence rates of pigmentary lesions in RRD eyes with flap tears, which may differ from those observed in typical LD, as pigmentary lesions (PLs) are often observed behind the retinal tears and may be located at the RPE or choroidal levels. Our preliminary observations suggest that this PL may coexist with typical LD in some cases. PLs not accompanied by LD may suggest a condition other than LD.

In the present study, we refer to this pigmented change observed in eyes with RRD and a flap tear as a pigmentary lesion (PL). This study aimed to elucidate the clinical features of PL and their associations with retinal flap tears and RRD in pre-, intra-, and post-operative imaging findings.

## Methods

This observational study was approved by the Institutional Review Board of the Kyoto University Graduate School of Medicine (Kyoto, Japan) and adhered to the tenets of the Declaration of Helsinki. Written informed consent was obtained at the initial visit from each subject prior to commencing the study. The study was performed in accordance with all the relevant guidelines and regulations.

This study included patients with unilateral RRD with PVD and retinal flap tears who visited the Department of Ophthalmology, Kyoto University Hospital, between January 2019 and February 2021. We excluded patients with RRD associated with atrophic hole, atopic dermatitis, trauma, macular hole, proliferative vitreoretinopathy, hereditary vitreoretinal degeneration (e.g., Wagner syndrome); patients with recurrent RRD; and patients with media opacities that made it difficult to observe the retinal features preoperatively. A total of 97 patients met the eligibility criteria.

UWF imaging of pre- and post-operative fundus color scanning light ophthalmoscopy (SLO) and green light fundus autofluorescence (FAF) was performed using an Optos 200T× imaging system or Optos Silverstone (Optos PLC, Dunfermline, UK). In addition, we reviewed intraoperative digital video recordings of vitrectomy using a non-contact 128 diopter front lens (Resight, Carl Zeiss Meditec AG) to evaluate the vitreous, retinal flap tears, and degeneration around the tears.

**Figure 1:**
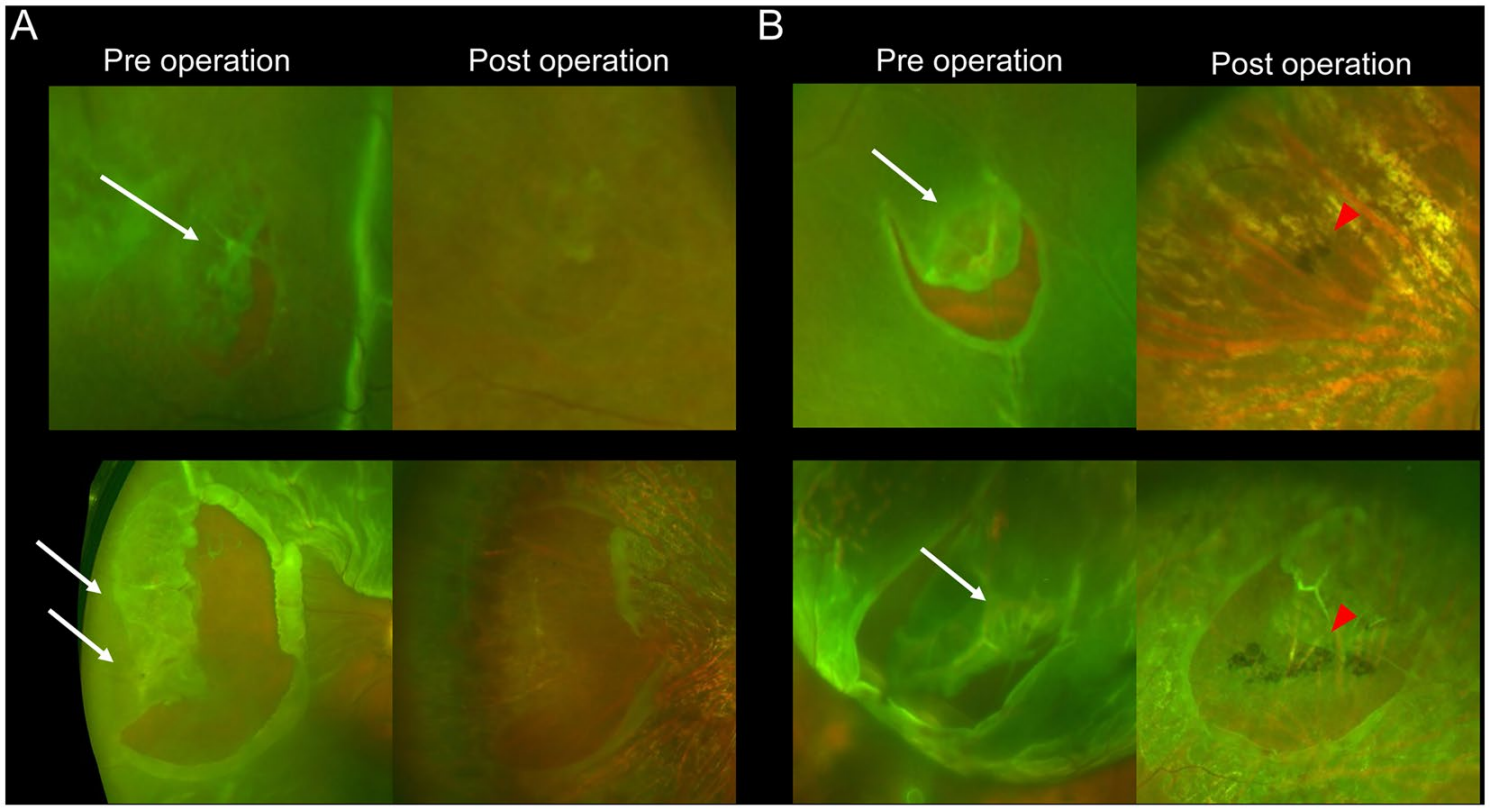
Lattice degeneration (LD) associated with rhegmatogenous retinal detachment (RRD) examined by pre- and postoperative ultra-widefield (UWF) color scanning light ophthalmoscopy (SLO). (**A**) LD without a pigmentary lesion (PL) in two representative cases. (**B**) LD with PL in two representative cases. The preoperative UWF images show the white vessels and retinal thinning within the LD lesions. White arrows indicate LD, and red arrowheads indicate PL.

We used the pre- and postoperative UWF SLO images and intraoperative digital video images to examine the structural features of the original flap tears and degenerative lesions. Based on previous reports, we defined LD as a circumferential and well-defined lesion in the retina located from the equator to its periphery, accompanied by the whitening of retinal vessels and thinning of the retina (Fig. 1).

**Figure 2:**
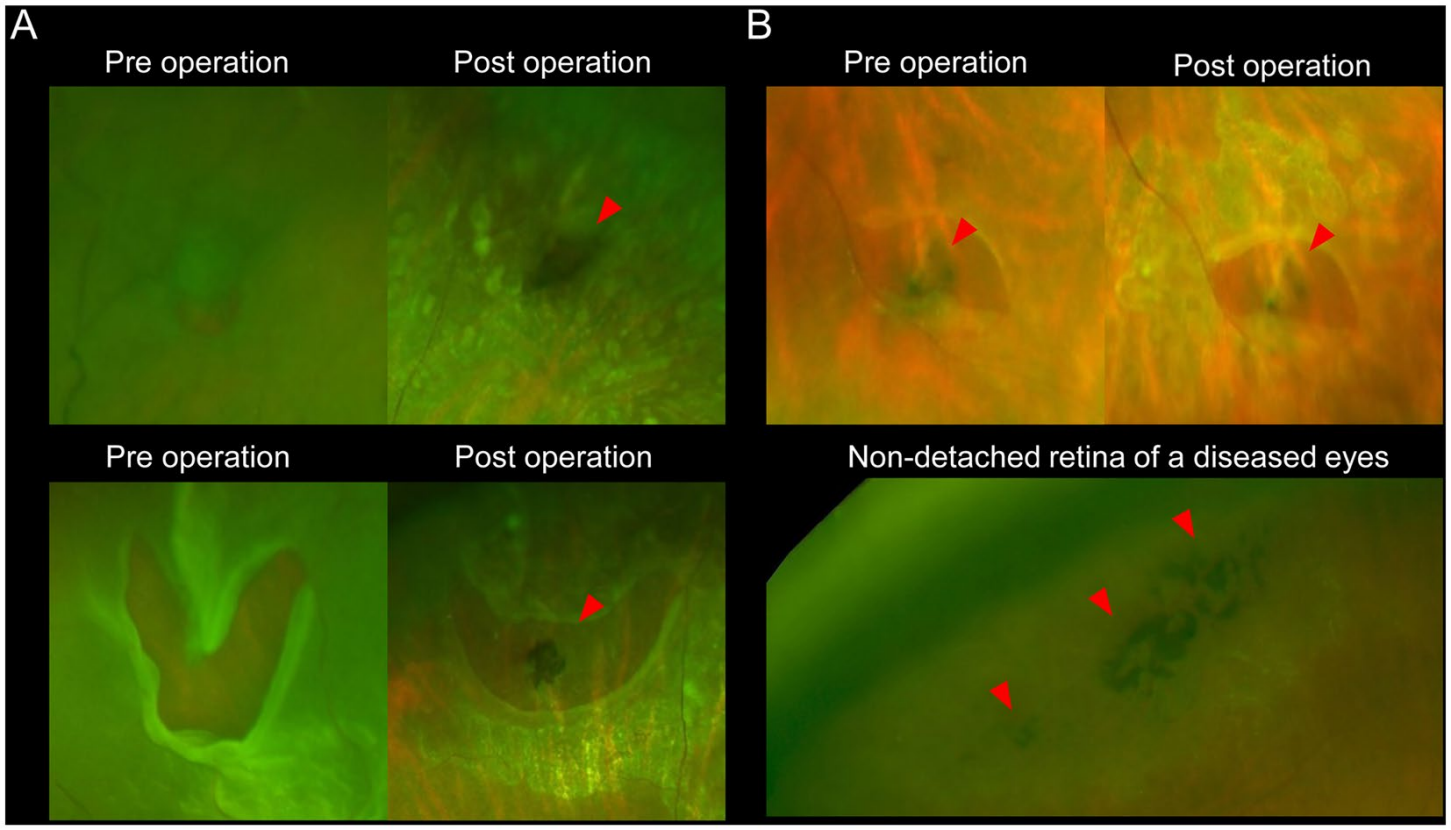
Pigmentary lesion (PL) of eyes with rhegmatogenous retinal detachment (RRD) examined by pre- and postoperative ultra-wide field (UWF) scanning light ophthalmoscopy (SLO). (**A**) Pigmentary lesion (PL) without lattice degeneration (LD) in representative two RRD-eyes. The preoperative UWF-SLO images do not clearly show the PL. However, the postoperative UWF-SLO images show the PL in and behind the retinal tears, at the retinal pigment epithelial level. In the eyes, the retinal tears do not accompany the LD lesion. (**B**) PL without LD in other RRD-eyes. The pre- and postoperative SLO images show the PL. Red arrowheads indicate PL.

We additionally defined PLs as a pigmented change in the deep retina or behind the retinal tears, reaching from the equator to the further periphery, that were distinct from typical LD (Fig. 2). LD or PL were classified independently by two retinal specialists (MI, KK). In cases of disagreement, the senior retinal specialist (YMu) made the final decision.

Preoperative examination of PLs (to which the RRD did not extend) in eyes with RRD was conducted using the Silverstone OCT (Optos PLC, Dunfermline, UK), Xephilio OCT-S1 (Canon Medical Systems, Japan), and Spectralis HRA + OCT (Heidelberg Engineering, Heidelberg, Germany) devices. These OCT devices and protocol used for examination (extended field imaging in Spectralis^31^) can capture wide-field images of the retina and depict equatorial retinal lesions.

### Statistical analysis

Statistical analysis was performed using PASW Statistics version 18.0 (SPSS, Chicago, IL). Values are presented as mean ± standard deviation. For statistical analysis, visual acuity (VA) values measured with a Landolt chart were converted to a logarithm of the minimum angle of resolution (logMAR). The Dunnett test was used to compare the respective VAs of eyes without both LD and PL, eyes with LD and without PL, eyes without LD and with PL, and eyes with both LD and PL.

## Results

This study included RRD patients with PVD and retinal flap tears. Table 1 shows the clinical characteristics of the included patients. The total sample size was 97 patients (women: 27, and men: 70), and the mean age was 56.8 ± 8.5 (range: 35–77) years. We performed pars plana vitrectomy (PPV; 87 eyes received 25 gauge PPV, and 10 eyes received 27 gauge PPV) for all eyes with RRD. Sulfur hexafluoride gas was used as the tamponade agent in 93 eyes and silicon oil was used in 4 eyes. All retinal tears were blocked intraoperatively using laser photocoagulation. The retinal reattachment rate was 100% (97eyes/97 eyes).

**Table 1:** Characteristics of patients with rhegmatogenous retinal detachment.

Total number of patients (women/men)	97 (27/70)
Mean age (years, range)	56.8 ± 8.5 (35–77)
Duration from onset of symptom (days)	11.0 ± 19.5
Visual acuity, logMAR	0.34 ± 0.62
Snellen visual acuity (range)	20/13–20/200
**Retinal detachment area, n (%)**	
< 1 quadrant	37 (38.1)
≥ 1 to < 2 quadrants	40 (41.2)
≥ 2 to < 3 quadrants	15 (15.5)
≥ 3 to ≤ 4 quadrants	5 (5.2)
Eyes with macula-off, n (%)	39 (40.2)
Number of retinal tears in the detached retina, n (range)	1.2 ± 0.49 (1–4)
Number of retinal tears outside of the detached retina, n (range)	0.12 ± 0.39 (0–2)
Mean axial length, (mm) ± S.D	26.3 ± 1.8 (22.8–30.8)
Phakia/pseudophakia, n	81/16

The data are shown as the mean ± standard deviation unless otherwise indicated.

We classified participants into the following 4 groups based the presence of PL and LD, and compared the preoperative and postoperative VA among the groups (Table 2). Eyes without both LD and PL were designated as the control group. The respective pre-, and post-operative VAs of other three groups were compared to those of the control group. The preoperative VA did not differ significantly among the groups, except the postoperative VA of the group with LD and without PL, which was significantly worse than that of the control group (*P* = 0.044).

**Table 2:** Preoperative and postoperative visual acuity based the presence or absence of lattice degeneration and pigmentary lesion.

	LD − , PL −	LD + , PL −	LD − , PL +	LD + , PL +	*P* *	*P* **	*P* ***
Preoperative VA (logMAR)	0.06 ± 0.41	0.41 ± 0.42	0.30 ± 0.70	0.45 ± 0.64	0.35	0.52	0.18
Postoperative VA (logMAR)	− 0.06 ± 0.11	0.13 ± 0.26	− 0.04 ± 0.14	0.05 ± 0.20	0.044	1.0	0.19

The VA values are presented as the mean ± standard deviation.

*VA* visual acuity, *logMAR* logarithm of the minimum angle of resolution, *LD* lattice degeneration .*PL* pigmentary lesion.

The Dunnett test was used to compare the respective VAs of eyes without both LD and PL, eyes with LD and without PL*, eyes without LD and with PL**, and eyes with both LD PL***.

There were 116 (mean count, 1.2 ± 0.5; range, 1–4 per eye) retinal tears in the detached retina of the included eyes. One-hundred and two (88%) of the retinal tears were accompanied by LD, PL, or another type of degeneration. A total of 63 (54%), 14 (12%), and 25 (22%) retinal tears were accompanied by both LD and PL, LD only, and PL only, respectively (Supplementary Table S1).

**Figure 3:**
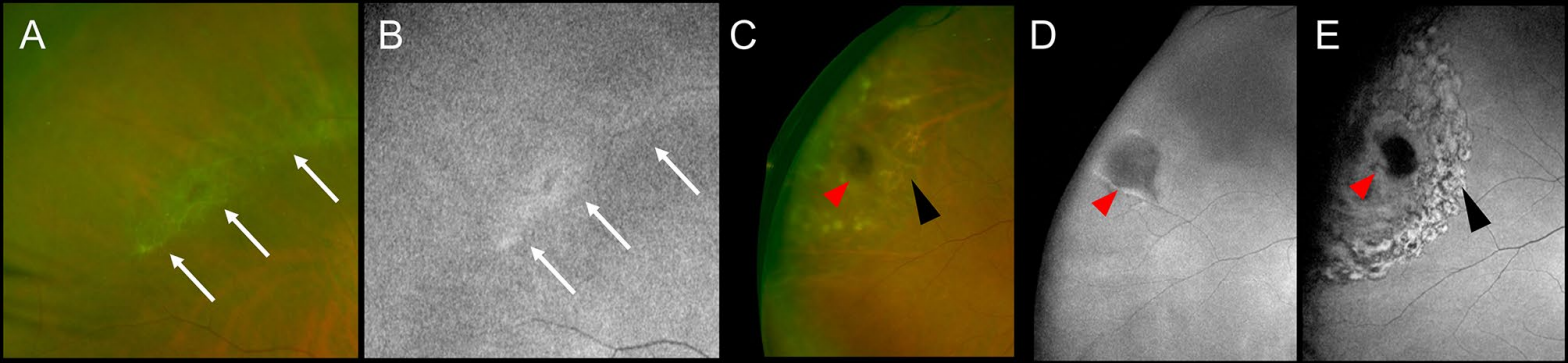
Green fundus autofluorescent (FAF) images of lattice degeneration (LD) and pigmentary lesion (PL). (**A**) and (**B**) LD in a non-detached retina of an eye with rhegmatogenous retinal detachment (RRD). The ultrawide field (UWF) scanning light ophthalmoscopy (SLO) image of LD (**A**). The green FAF shows normal to mild-hyper fluorescence in the LD lesion (**B**). White arrows indicate LD. (**C**)–(**E**) The PL seen in an original flap tear of another RRD-eye. The postoperative UWF SLO image shows the PL and retinal photocoagulation (PC) scars (**C**). The pre- and post-operative green FAF images of the PL in the original retinal flap tear, respectively (**D**, **E**). The PL shows hypofluorescence, which is distinguished from the appearances of the laser scars around the retinal tear. Red arrowheads indicate PL. Black arrowheads indicate PC scars.

In green FAF images, LD lesions showed normal to mild-hyper fluorescence. In contrast, all PLs showed hypofluorescence (Fig. 3).

**Figure 4:**
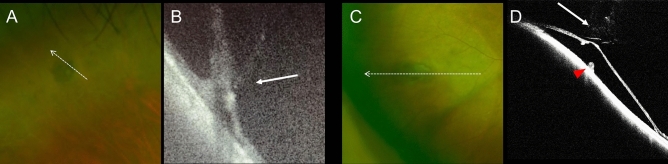
Optical coherence tomography (OCT) images of pigmentary lesion (PL) at retinal areas that are not affected by rhegmatogenous retinal detachment (RRD). (**A**) The ultrawide field (UWF) scanning light ophthalmoscopy (SLO) image of PL. This retinal area is not affected by the RRD. (**B**) The OCT image of PL along the dotted arrow. Vitreous traction to the PL is seen. (**C**) The UWF SLO image of PL of other RRD-eyes. This retinal area is not affected by the RRD. (**D**) The OCT image of the PL along the dotted arrow. In addition to the vitreous traction (arrow), tractional retinal detachment and elevation of the retinal pigment epithelial level (red arrowhead) are seen.

On OCT examination, PLs were located at the RPE level, which was elevated. PL-associated abnormalities were unclear on the OCT images that segmented the choroid. In the retinal areas of 22 eyes that were not affected by RRD, we observed PLs without obvious retinal tears. Some of the PLs were accompanied by vitreous traction and tractional retinal detachment (Fig. 4).

## Discussion

This study aimed to examine the characteristics of PL observed in eyes with RRD and a flap tear. The pre- and post-operative UWF imaging and intraoperative direct observation of eyes with RRD suggested that PLs were frequently present at the site of the original flap tear, which tended to occur at the level of the RPE and behind the retinal tear.

Retinal detachment is a condition in which the sensory retina detaches from the RPE. Depending on the underlying causes, retinal detachment can be divided into serous (exudative) and tractional retinal detachments, and RRD. RRD is associated with the formation of retinal holes or tears, and it has an incidence of approximately 1 per 10,000 persons per year. Retinal holes generally occur on the atrophic peripheral retina of myopic eyes and are weakly associated with PVD. In contrast, retinal flap tears occur with the development and extension of PVD. The PVD itself is an age-dependent physiological phenomenon. However, in pathologic areas with abnormal adhesions to the retina, the retina is pulled anteriorly during the progression of PVD, resulting in the formation of retinal tear, and RRD^6,7^.

The LD of the retina is considered a typical lesion associated with RRD. The rate of LD-related RRD has been previously reported in the range of 46.8–65.7% in Japan^4,21^, which was higher than the estimates reported for Europe and the United States. This discrepancy may be accounted for by the fact that myopia is more prevalent in Asia, including Japan, than elsewhere. In the present study, LD was found in 77 (66.4%) of 116 original retinal tears. The results of this study were consistent with those of previous studies in the Japanese population^4,23^. The LD is ophthalmoscopically characterized by circumferential degeneration of the retina with clear borders accompanied by vascular whitening and thinning of the retina^16,17^. The pathological features of LD are mainly seen within the retina or vitreoretinal interface, and include the absence of the inner limiting membrane, abnormality of retinal vessels^16^, disruption of the layered-structure^16^, replacement of retinal tissue by glia cells^16^, liquefaction of the nearby vitreous^25^, and strong vitreous adhesion at the degeneration edge^25^. Pathological changes to the RPE in LD remain unclear, which was not consistent with the presence of RPE findings in PL of the current study.

In this study, we examined the characteristics of PLs in eyes with flap tear-associated RRD based on pre-, intra-, or postoperative observations. However, we did not include RRD eyes without retinal flap tear, since most cases of RRD without flap tear are considered to be the result of retinal atrophic holes, which are commonly seen in the eyes of young patients with high myopia. Since these eyes are generally not treated with intraocular surgery (vitrectomy) but with extraocular buckling surgery, intraoperative observation is not applicable for these eyes. Hence, we excluded eyes with RRD caused by retinal hole from this study, which did not allow us to compare the frequency of PL between RRD eyes with and without flap tears. We endeavor to examine these aspects in a future study.

The postoperative VA was significantly worse in the group with LD and without PL compared to the group without both LD and PL. However, the relationship between the presence of LD or PL and postoperative VA is unclear. In general, we believe that VA in eyes with RRD is greatly influenced by whether or not the retinal detachment involves the macula.

In the present study, PL was found in more than 75% of all original tears and was co-localized with LD in 54% of all flap tears. This high rate of co-localization suggests that PL may be associated with LD. However, LD and PL were found alone in 12% and 22% of the patients, respectively, suggesting that they may be separate pathologies. The depth of PL may have reached the level of the RPE and/or choroid because the lesion was intraoperatively observed through the retinal tears and on the RPE surface. However, our OCT examination did not clearly show whether the lesion involved the choroid because the pigment of the lesions obscured the boundary between the RPE and choroid. On green FAF images, the LDs appeared as normal to slightly hyperfluorescent structures, whereas PLs appeared as hypofluorescent structures in all cases, suggesting that the two entities show different properties on FAF imaging. Overall, it is likely that the low fluorescence of PL was not due to the RPE atrophy, but rather due to the melanin pigment blocking the fluorescent substance. In summary, in this study, PL was a pigmented change located at the site of the original retinal tear and present at the RPE level, and with some features distinct from those of LD.

In this study, we obtained preoperative OCT images of PLs observed in retinal areas that were not affected by RRD. These images revealed that PL was occasionally accompanied by vitreous traction and tractional retinal detachment (a tear was not evident) (Fig. 4). We speculate that PL may have caused the vitreous adhesion and tractional retinal detachment, which in turn caused retinal flap tears and RRD. However, PL may be a secondary change that occurs after retinal detachment. Although laser photocoagulation performed for the retinal tears can cause pigmentary change around the PL, we could differentiate between the PL and the laser photocoagulation scars because we evaluated the PL preoperatively and intraoperatively. Additionally, we could also differentiate the PL from the laser photocoagulation scars postoperatively (Fig. 3). In cases where retinal detachment resolves spontaneously, we occasionally observe pigmentary changes around the edge of the detachment area^16^. However, serous and tractional retinal detachments are unlikely to accompany pigmentary changes; thus, it is unlikely that the observed PL was secondary to RRD. Given the high frequency of PL at the original tear sites and occasional co-occurrence of PL and tractional RD along with vitreous traction, PL might be a risk factor for RRD.

This study has some limitations. First, it was a retrospective observational study. Consequently, it remains unclear what percentage of the general population is affected by PL, and how many patients with PL progress to RRD. Second, although we used color SLO and intraoperative video findings to distinguish LD and PLs based on previous findings, the classification remains subjective. In addition, because we observed the RPE lesion through the retinal flap tears, we could not comprehensively evaluate the RPE, including the retinal areas that were not detached. Finally, because the RRD patients were often seen outside of office hours, OCT imaging of the retinal flap tears was not always performed. However, despite these limitations, the present study suggests that PL may present at RPE levels behind the original tears in eyes with RRD. These findings suggest that PL might be a risk factor for RRD, developing alongside or separately from LD. Prospective studies are required to validate these findings.

## Supplementary Information

Supplementary Table 1.

## Data Availability

The datasets generated during and/or analyzed during the current study are available from the corresponding author on reasonable request.
