# Supplementary material for: Pigmentary lesions in eyes with rhegmatogenous retinal detachment with flap tears: a retrospective observational study
Source: Sci Rep. 2022 Jul 21;12:12470. doi: 10.1038/s41598-022-16508-5 (PMC9304380; doi:10.1038/s41598-022-16508-5)
Supplement: Supplementary file 1 — Supplementary Table 1. [file 41598_2022_16508_MOESM1_ESM.docx]

| **Supplementary Table 1.** Locations of lattice degeneration and pigmentary lesions | | | | | |
| --- | --- | --- | --- | --- | --- |
|  | LD | PL | Both | Other | Total |
| Detached retina | n (%) | | | | |
| Superotemporal | 7 (50.0) | 14 (56.0) | 35 (55.6) | 3 (21.4) | 59 (50.9) |
| Superonasal | 4 (28.6) | 5 (20.0) | 15 (23.8) | 10 (71.4) | 34 (29.3) |
| Inferotemporal | 2 (14.3) | 3 (12.0) | 11 (17.5) | 1 (7.1) | 17 (14.7) |
| Inferonasal | 1 (7.1) | 3 (12.0) | 2 (3.2) | 0 (0) | 6 (5.2) |
| Total | 14 (100) | 25 (100) | 63 (100) | 14 (100) | 116 (100) |
|  | LD | PL | Both |  | Total |
| Non-detached retina | n (%) | | | | |
| Superotemporal | 3 (21.4) | 2 (22.2) | 6 (21.4) |  | 11 (21.6) |
| Superonasal | 2 (14.3) | 3 (33.3) | 6 (21.4) |  | 11 (21.6) |
| Inferotemporal | 5 (25.7) | 3 (33.3) | 9 (32.1) |  | 17 (33.3) |
| Inferonasal | 4 (28.6) | 1 (11.1) | 7 (25.0) |  | 12 (23.5) |
| Total | 14 (100) | 9 (100) | 28 (100) |  | 51 (100) |
| LD = lattice degeneration, PL = pigmentary lesion. | | | | | |
